# Synbiotics in caries prevention: A scoping review

**DOI:** 10.1371/journal.pone.0237547

**Published:** 2020-08-12

**Authors:** Mohammed Nadeem Bijle, Manikandan Ekambaram, Edward C. M. Lo, Cynthia Kar Yung Yiu

**Affiliations:** 1 Paediatric Dentistry, Faculty of Dentistry, The University of Hong Kong, Hong Kong, Hong Kong; 2 Paediatric Dentistry, Faculty of Dentistry, University of Otago, Dunedin, New Zealand; 3 Dental Public Health, Faculty of Dentistry, The University of Hong Kong, Hong Kong, Hong Kong; Oregon Health & Science University, UNITED STATES

## Abstract

The scoping review aimed to examine the evidence on the role of synbiotics in caries prevention. PubMed, SCOPUS, and Web of Science databases search were performed. Any *in vitro* study, clinical trial, systematic review with/without meta-analysis, umbrella review/meta-evaluation, narrative review addressing the role of synbiotics in caries prevention were included in the scoping review. Data were extracted from the included studies using pre-approved registered protocol. Twenty-eight records were identified, of which 5 *in vitro* studies, 1 quasi-experimental clinical trial and 1 narrative review were included in the present review. No controlled clinical trials or systematic reviews on the role of synbiotics in caries prevention could be identified. Except 1, all *in vitro* studies examined the combined effect of saccharides and lactobacilli spp. as potential synbiotics on the growth of *Streptococcus mutans*. However, the proposed synbiotics in 4 *in vitro* studies either did not qualify or remained ambiguous of its eligibility as a potential synbiotic for caries prevention. One recent *in vitro* study explored the possibility of L-arginine and *Lactobacillus rhamnosus* GG synbiotic for caries prevention. The quasi-experimental clinical study without a control arm did not explicitly mention the intervention composition and thus, its synbiotic potential remains unclear. A narrative review highlighted the potential of combining arginine (prebiotic) with arginolytic bacteria (probiotic) as a synbiotic, which appears promising for caries prevention. The eligibility of the proposed synbiotics as a true synbiotic needs to be carefully addressed. Due to a lack of controlled clinical studies on synbiotics for caries prevention, evidence on their caries-preventive potential is weak. Future studies are needed to examine the combination of amino acids (esp. arginine) with probiotics as a potential synbiotic against cariogenic pathogens.

## Introduction

Global Burden of Disease Study in 2016 estimated that 2.4 billion people suffer due to dental caries of permanent teeth worldwide, with an additional 486 million children suffer from caries of primary teeth [1]. Dental caries negatively influences oral-health related and psychosocial quality of life [2–4]. Besides detrimental impact on quality of life, extra financial burdens are imposed for treatment of dental caries and subsequent maintenance. Therefore, prevailing caries prevention is inevitable to reduce the global burden of caries and associated negative impacts.

Dental caries is a chronic dysbiosis-associated biofilm disease that manifests when the cariogenic pathogens dominate over the healthy oral commensals. Metabolism of fermentable carbohydrate by the cariogenic pathogens leads to prolonged acidic microenvironments in the tooth-adhered biofilm. Demineralization of the tooth hard tissues occurs below the critical pH when the net mineral loss is higher than the mineral gain [5]. Thus, contemporary dental caries prevention and management should include a two-prong strategy for regulating the pathogenic biofilms and restoring mineral loss. Regular use of fluoride toothpaste has led to a remarkable decline in the dental caries worldwide [6], as it substantiates continuous availability of low levels of fluoride in the oral cavity, which inhibits enamel demineralization and promotes remineralization [7]. However, fluoride offers limited biofilm control [8]. Thus, for effective caries control, it is imperative to supplement fluoride with biofilm-targeted strategies that aid to reverse the microbiome dysbiosis.

Biofilm-targeted strategies for restoring ecological symbiosis can either be biofilm inhibitory or biofilm modulating. Inhibition of bacterial biofilm using antimicrobial agents raises the concern of antimicrobial resistance in the long term, which escalates the negative impact on oral microbial ecology. Oral biofilm homeostasis is a key component of maintaining oral health and preventing disease [9]. Therefore, the biofilm modulating approach that restores or maintains homeostasis in the oral cavity is desirable for caries prevention. Biofilm modulation aims to enhance the growth of healthy oral commensals, effectively attenuating the presence of pathogens, thereby maintaining a diverse symbiotic ecological microcosm. The modulation can either be triggered by stimulating the metabolic activities of commensals or external supplementation of beneficial probiotics.

Prebiotics and probiotics are well known for their beneficial effects on the health of the human gastrointestinal tract. Prebiotic was first defined by Gibson and Roberfroid as “a non-digestible food ingredient that beneficially affects the host by selectively stimulating the growth of bacteria, and thus improves host health” [10]. Oral prebiotics such as arginine have been extensively studied in the current decade for caries prevention with synthesized and appraised evidence from clinical trials [11,12]. Although the synthesized evidence with arginine-containing products showed superior caries-preventive effect than controls, the appraisal had raised concerns on research ethics of clinical trials. Furthermore, long-term use of arginine poses a risk of increased plaque alkalization and overgrowth of oral anaerobes such as *Porphyromonas gingivalis* [13].

Another N-containing compound–carbamide, has been studied for its anti-caries prebiotic effect; however, the clinical trials showed no significant difference in the anti-caries effect between the carbamide-based intervention and control [14,15]. Recently, two potential prebiotics (β-methyl-D-galactoside and N-acetyl-D-mannosamine) were identified for the oral cavity which selectively triggered commensal bacteria *in vitro* demonstrating a biofilm shift with dominance of commensals [16,17]. However, their effect in caries prevention is yet to be discerned.

As per WHO, probiotics are defined as “live micro-organisms which when administered in adequate amounts confer a health benefit on the host” [18]. Similar to prebiotics, several clinical studies examining the effect of probiotics on caries prevention have been conducted in the last two decades using different probiotics strains and species in several deliverable forms [19]. Lactobacilli and bifidobacteria probiotics have been extensively studied in clinical trials with empirical caries-preventive effect; however, probiotic colonization in the oral cavity is transient. Therefore, formulations enhancing probiotics survival are needed to impart long-standing caries-preventive benefits.

Synbiotics are defined as “mixtures of probiotics and prebiotics that beneficially affects the host by improving the survival and implantation of live microbial dietary supplements” [20]. The development of synbiotics may be more efficient in the prevention of dental caries than the use of either one alone as the mixture could compensate for the drawbacks of the individual components while imparting a synergistic caries-preventive effect. The evidence of prebiotics and probiotics on dental caries prevention has been separately reported in clinical trials and summarized in several systematic reviews [11,12,19]. However, until now the role of synbiotics in caries prevention is currently unclear. A preliminary search of published studies examining the effect of synbiotics on caries prevention showed relatively broad heterogeneity in design and quality; therefore, a systematic review was inappropriate. Instead, we performed a scoping review to critically review the evidence of synbiotics on caries prevention, addressing any research gaps to inform the design for future investigations.

## Methods

The present study is reported as per PRISMA-ScR guidelines (S1 Table) and previous reports on scoping review methodology [21–23]. A previous report is the seminal paper [21] on the scoping reviews, which formed the basis for conducting the present study.

### Study protocol

The study protocol was prepared prior to conducting the study selection and registered on Open Science Framework for public view with the accession link - https://osf.io/srdc5/. The protocol was drafted using PRISMA-ScR guidelines and included the following sections–study aim, search strategy, eligibility criteria, outline for data summary charting as per consensus by all the contributors of the study.

### Inclusion criteria

Any *in vitro* study, clinical trial, systematic review with/without meta-analysis, umbrella review/meta-evaluation, narrative review addressing the role of synbiotics in caries prevention were included in the scoping review.

### Exclusion criteria

Studies with no keywords specific components, commentaries, opinions, and articles in languages other than English were excluded from the review. Articles addressing the role of probiotics (only) or prebiotics (only), without including synbiotics in caries prevention, as well as those addressing the role of synbiotics on other oral diseases, like oral candida infections or periodontitis were also excluded from this review.

### Database search strategy

The articles were identified for inclusion in the review from the databases PubMed, Scopus, and WoS. Additionally, google search was performed and expert opinion was sought (CKYY, ECML) for identifying articles with the final refined search strategy. Further, references of the identified articles were hand searched prior to initiating the screening process to identify potential records for inclusion in the review. All possible search using the search strategy was conducted until 17 July 2020.

The search strategy included the use of wildcard (*) for the keyword “synbiotics” whereby “synbiotic” was limited and the wild card specified any further number of characters for search by the databases. Then, a Boolean operator “AND” was incorporated in the database search to connect the keyword “caries”. The keyword “prevention” was not included in the search so as to identify a larger number of records on synbiotic and caries using the most significant keywords. The detailed search strategy for individual databases is shown in S2 Table.

### Study selection and data charting

Irrespective of the study design, peer-reviewed articles in English that fulfilled the inclusion/exclusion criteria, addressing the role of synbiotics in caries prevention were eligible for inclusion. The base map for final inclusion was—Population-(any)-concept-(synbiotics)-context-(caries prevention). No specific primary or secondary outcome measure(s) were defined for the review. The study selection and data charting process were performed by two independent reviewers (MNB and ME). Disagreements in the process were resolved by seeking expert advice from two other reviewers (CKYY and ECML). The articles for final inclusion in the review were as per the consensus of all the reviewers.

The data charted for the included studies was as per the pre-approved registered protocol. The variables extracted to chart the data were author (year), location, study type and objectives, results and conclusion, and inferred limitations. The charted data was approved and confirmed by all the reviewers prior to summarising the results. Data synthesis for the scoping review was planned on multiple controlled clinical studies included in the review.

## Results

### Evidence selection

**Fig 1:**
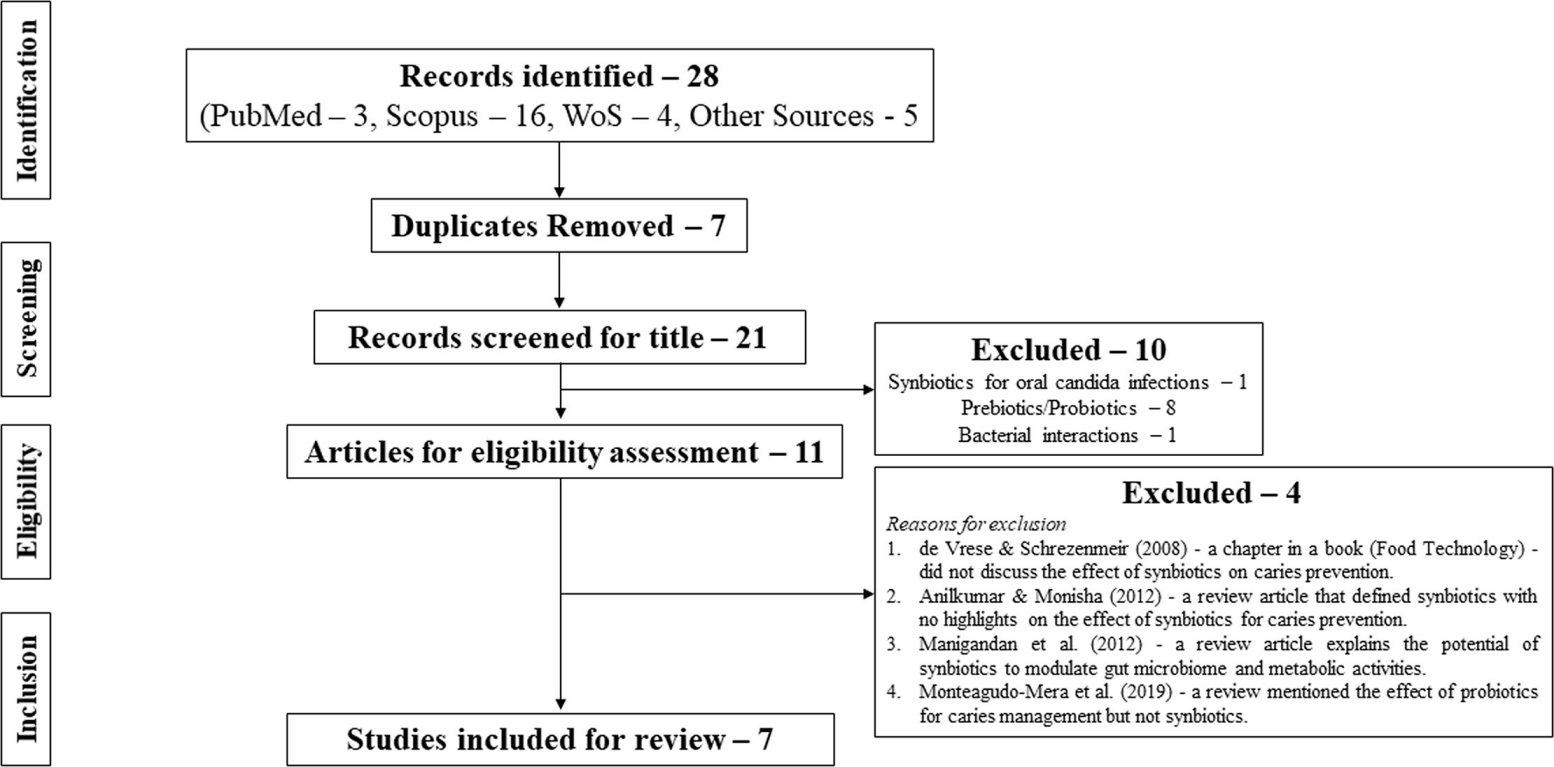
Study selection process.

The flow of the evidence selection in the present review is shown in Fig 1. Twenty-seven records were identified from 3 databases and other sources (expert opinion by CKYY, ECML and google search). From the search of reference lists of identified records, 1 additional study was identified for possible inclusion. The study was retrieved for screening and eligibility assessment. Therefore, a total of 28 records were identified, of which 7 were duplicates. Twenty-one records were screened for title and abstract content; of which 10 [24–33] were excluded. Of the excluded studies, 1 study was a mini-review that discussed the potential of synbiotics for oral candida infections [24], 8 studies were on prebiotics/probiotics (alone or both discussed as an individual intervention) [25–32], and another study explored the polymicrobial interactions in pathogenicity [33]. Full-text of 11 articles were reviewed for eligibility assessment and eventually 7 [34–40] were included in the review based on the inclusion/exclusion criteria. The reasons for excluding 4 articles [41–43] are presented in the Fig 1.

### Study characteristics

The study characteristics of the included publications are presented in Table 1. Of the 7 included articles in the review, 5 [34,35,37–39] were *in vitro* studies, 1 article was a quasi-experimental clinical trial [40]; while 1 article [36] was a narrative review. No controlled clinical trials or systematic reviews on the role of synbiotics in caries prevention could be identified. Of the 5 *in vitro* studies, 2 studies [35,37] were preliminary reports examining the effect of synbiotics and their effects on cariogenic bacterium *Streptococcus mutans*. One study was from the UK [37], 1 from Japan [38], 1 from Hong Kong [39], and 2 studies [34,35] were from Thailand. One clinical trial was from Mexico [40] and a narrative review [36] was from Europe. It is noteworthy that all the studies were published in the current decade (2010 onwards) emphasizing that the concept of synbiotics in caries prevention is relatively recent.

**Table 1:** Study characteristics of the included publications.

SN	Author (Year)	City, Country	Study Type	Study Objectives	Results and Conclusions	Limitations
1	Tester & Al-Ghazzewi (2011)	Glasgow, UK	*In vitro* study	To determine the effect of *L*. *acidophilus* (probiotic) and glucomannan hydrolysate (prebiotic) on the growth of *S*. *mutans*.	*Lactobacillus acidophilus* outgrew *S*. *mutans* in the presence of glucomannan hydrolysate. The study concluded that the proposed synbiotics can potentially improve the ecology of mouth by suppressing the oral pathogens.	The article was a preliminary *in vitro* study whereby the mechanistic of the proposed synbiotics was not explained. In addition, only 1 concentration (2%) of the prebiotic was tested against the control without appropriate rationale for such a comparison.
2	Kojima et al. (2016)	Kanagawa, Japan	*In vitro* study	To discover probiotic and prebiotic candidates that can be developed as a novel synbiotics against bacterial and fungal pathogens.	The study identified arabinose, xylose, and xylitol as suitable prebiotics and five lactobacilli strains isolated from oral cavity as suitable probiotics; whereby, the lactobacilli strain had an inhibitory effect on the production of insoluble glucans by *S*. *mutans*. The combined prebiotics and probiotics can be developed as a suitable synbiotics to incorporate in oral healthcare products that can neutralize the growth of oral pathogenic microorganisms without disrupting the balance of healthy environment.	Optimal combinations of saccharides and lactobacilli needs to be elucidated and the active molecules that mediate the inhibitory effect of lactobacilli should be identified.
3	Nunpan et al. (2017)	Phitsanulok, Thailand	*In vitro* study	To investigate the inhibitory effect of synbiotics between galacto-oligosaccharides and *L*. *acidophuilus* (TISTR 2365T = DSMZ 20079T) on the growth of *S*. *mutans* (DSMZ 20523T)	The galacto-oligosaccharides did not enhance the function of *L*. *acidophilus* to inhibit the growth of *S*. *mutans*.	The findings was from a preliminary study whereby the proposed synbiotics showed some effects against *S*. *mutans* but the effect was non-significant.
4	Nunpan et al. (2019)	Phitsanulok, Thailand	*In vitro* study	To investigate the effect of probiotics (*L*. *acidophilus* ATCC 4356 or DSMZ 20079) on *S*. *mutans* (A-32) after the probiotics received the prebiotics (galacto-oligosaccharides and fructo-oligosaccharides).	The prebiotics—galacto-oligosaccharides and fructo-oligosaccharides, had no effect on the growth of *L*. *acidophilus* (probiotic), and the 3% fructo-oligosaccharides significantly decreased the growth rate of *S*. *mutans*. The 3% galacto-oligosaccharides and 1% fructo-oligosaccharides seem to have the potential to decrease the growth rate of *S*. *mutans* when applied together with the probiotics (*L*. *acidophilus*).	The study authors did not identify the mechanistics of the proposed synbiotics for its effect against *S*. *mutans*.
5	Zaura & Twetman (2019)	Copenhagen, Denmark	Narrative review	To discuss the current evidence for the role of oral prebiotics and probiotics in caries prevention and caries management, as well as the future directions in the field.	The development and evaluation of oral synbiotic products would be of interest in the future management of dental caries. Combining prebiotic arginine with arginolytic probiotics may be a future synbiotic for the prevention and management of dental caries.	The review article authors suggest the possibility of arginine and arginolytic probiotics as a future synbiotics for caries prevention; however, the development and evaluation of such a combination is needed.
6	Hernandez et al. (2020)	San Luis, Mexico	Quasi-experimental clinical trial	To evaluate the effect of a synbiotic (Lactiv®) on salivary viscosity (primary outcome) and buffer capacity (secondary outcome).	The synbiotic use over a period of 6 days significantly decreased the salivary viscosity and improved saliva buffer capacity of 24 individuals with active tooth decay.	The authors of the study did not include any control arm (as per the study design) and the patient recruitment was based on consecutive-convenience sampling.
7	Bijle et al. (2020)	Hong Kong, SAR China	*In vitro* study	To examine the effect of L-arginine on the growth of *L*. *rhamnosus* GG and combined *L*. *rhamnosus* GG and L-arginine synbiotic on the growth of *S*. *mutans*	Increasing concentrations of L-arginine enhanced the growth of *L*. *rhamnosus* GG. The synbiotic (L-arginine at 2% *w/v*. + *L*. *rhamnosus* GG) significantly inhibited the growth of *S*. *mutans*. The proposed synbiotic has a significant potential to develop as an anti-caries regimen.	The study contributors used a mono-species closed microbial biofilm model as opposed to open model. The effect of the promising synbiotic can be further explored in future studies using polymicrobial open biofilm model.

Except one, all *in vitro* studies [34,35,37,38] examined the combined effect of saccharides and lactobacilli spp. on the growth of *S*. *mutans*. Three *in vitro* studies [34,35,37] utilized *Lactobacillus acidophilus* as a probiotic in their proposed synbiotics. One recent *in vitro* study explored the possibility of L-arginine and *Lactobacillus rhamnosus* GG synbiotic for caries prevention [39].

A quasi-experimental clinical trial, included a synbiotic intervention (unclear of its prebiotic component) with multi-species probiotics [40]. The narrative review [36] highlighted the potential of combining the prebiotic arginine with probiotic arginolytic bacteria (e.g. *Streptococcus dentisani and Streptococcus A12*) and develop a synbiotic for caries prevention, based on the evident effect of arginine against caries as shown by the primary studies included in the review. However, such an arginine-based synbiotic is yet to be developed, implemented, and/or further assessed in laboratory and clinical studies.

### Evidence insights on synbiotics for caries prevention

The included articles in the review are further discussed in the following sub-categories based on their proposed synbiotics.

### Saccharides and lactobacilli spp. or multi-species synbiotics

The first preliminary report, unveiling the caries-preventive potential of synbiotic by examining its effect of on the growth of *S*. *mutans* was published in 2011 [37]. The study evaluated the effect of a synbiotic combination of *L*. *acidophilus* NCFB 1748 (probiotic) with glucomannan hydrolysate (GMH), which is a polysaccharide to stimulate the growth of probiotic microorganisms. The *L*. *acidophilus* and *S*. *mutans* were co-cultured in modified Columbia base broth supplemented with 2% GMH for 72 h and then plated using spread plate technique for 48 h to determine the growth of bacteria (*S*. *mutans* and *L*. *acidophilus*) compared to the respective controls. Results of the study showed that after incubation, *L*. *acidophilus* outgrew *S*. *mutans* in the presence of GMH. The study concluded that the consumption of GMH as a prebiotic by the probiotic (*L*. *acidophilus*) can potentially improve the ecology of the mouth by promoting the growth of beneficial bacteria and suppressing pathogens. The synbiotic could potentially be used as a prophylactic or therapeutic agent for reducing the presence of pathogens associated with tooth decay and be further developed for applications in oral health. However, the preliminary study did not outline the mechanistic properties of the proposed synbiotic. Furthermore, only 1 concentration (2%) of the prebiotic was tested against the control without providing appropriate rationale for such a comparison.

The second study [38], which was conducted in Japan, attempted to develop new synbiotics against oral pathogens (bacterial and fungal). Prebiotic screening was conducted by a sugar assimilation test using 12 saccharides; while 40 strains of lactobacilli (22 strains isolated from human oral cavity, 11 strains from dairy products and 7 reference strains) were used for probiotic screening. The amount of insoluble glucan produced by *S*. *mutans* was determined using phenol-sulphate staining as a growth inhibitory test. Five strains (*L*. *gasseri*, *L*. *fermentum*, *L*. *paracasei*, *L*. *plantarum*, and 120. *Lactobacillus* spp.) isolated from human oral cavity inhibited insoluble glucan production by *S*. *mutans*. In contrast, the lactobacilli strains isolated from the dairy products did not show an inhibitory effect on oral pathogens. Saccharide assimilation test showed that *S*. *mutans* growth was inhibited in the presence of xylose, xylitol, and arabinose, which demonstrated strong prebiotic potential. The study revealed that not only live lactobacilli probiotic bacteria, but lactobacilli with culture supernatant were also capable of inhibiting *S*. *mutans*. The study identified potential prebiotics and probiotics that could be further developed as suitable oral synbiotics for incorporation into oral healthcare products. However, optimal combinations of saccharides and lactobacilli that can be employed for clinical use still need to be determined and the mechanistic properties of synbiotics should also be identified.

Another preliminary study [35] evaluated the efficacy of the galacto-oligosaccharides (GOS) to enhance the probiotic (*L*. *acidophilus* TISTR 2365T or DSMZ 20079T) for inhibition of *S*. *mutans* DSMZ 20523T. The efficacy of the synbiotic against *S*. *mutans* was determined by assessing their growth rates using CFU assay. The concentrations of GOS used in the study were 1%, 2%, 3% & 4% *v/v*. The growth rate of *S*. *mutans* increased in the presence of 1% and 2% GOS; while the growth rate of *S*. *mutans* decreased with 3% and 4% GOS compared to the control group. The growth rate of *L*. *acidophilus* increased with 1% GOS supplementation; whereas, with 2% GOS the growth of *L*. *acidophilus* decreased and relatively constant growth rate of *L*. *acidophilus* was seen with 3% and 4% GOS. The study concluded that GOS was not an efficient prebiotic and did not enhance the function of the probiotic (*L*. *acidophilus*) to inhibit the growth of *S*. *mutans*.

Another reported *in vitro* study [34] evaluated the efficacy of prebiotics GOS and fructo-oligosaccharides (FOS) for enhancing the growth of probiotic *L*. *acidophilus* ATCC 4356 (DSMZ 20079T) and the combined pre- and pro-biotics for inhibiting *S*. *mutans* A32-2 (a clinically isolated strain from patients with high active caries). The studied GOS concentrations were 1%, 2%, 3%, 4% and 5% *v/v*; while FOS concentration was 1%, 2%, 3%, 4% and 5% *w/v*, respectively. The growth rates of *S*. *mutans*, *L*. *acidophilus* or combined *S*. *mutans* and *L*. *acidophilus* was determined at 3, 6, and 12 h after culture or co-culture. The study showed that *S*. *mutans* growth significantly decreased when co-cultured with *L*. *acidophilus* in the 3%, 4%, and 5% GOS supplemented medium. Further, with the FOS supplemented medium, the growth rate of *S*. *mutans* was significantly inhibited at all concentrations when co-cultured with *L*. *acidophilus*. There was no reported significant difference in the growth rate of *L*. *acidophilus* in all concentrations of GOS- and FOS- supplemented media as compared to the control.

Furthermore, the effect of the prebiotics on the cellular fatty acids and the secreted fatty acids were determined for *S*. *mutans* and *L*. *acidophilus* separately and co-cultured. The percentage of cellular fatty acids and secreted fatty acids, individually grown or co-cultured in the prebiotics-supplemented culturing medium were similar to the control group. The influence of the prebiotics supplementation on pH of the medium was evaluated. It was found that the pH of the FOS- and GOS-supplemented medium was similar for both *S*. *mutans* and *L*. *acidophilus*; whereas the pH with individual cultured *S*. *mutans* was higher than that of either the individual *L*. *acidophilus* culture or the co-culture. The study concluded that the 3%, 4% and 5% GOS and 1% FOS with *L*. *acidophilus* could be potential synbiotics against *S*. *mutans*. However, the study did not identify the mechanistic properties of the proposed synbiotics against cariogenic pathogen *S*. *mutans*.

A quasi-experimental clinical study [40] was performed on 24 individuals (16 females, 8 males) with mean age of 10.92 years and active tooth decay. In this study, the effect of a commercial synbiotic (Lactiv®, Teva Termekek, Budapest, Hungary) primarily indicated for gut microflora, on salivary viscosity (primary outcome) and saliva buffer capacity (secondary outcome), as biomarkers indicating caries-preventive potential was evaluated. The primary outcome variable was assessed using a calibrated Ostwald Pipette method and the secondary outcome measure was determined using bromocresol blue-HCl method. After 6-day daily synbiotic intake, it was observed that the salivary viscosity decreased significantly compared to the baseline data, while the saliva buffer capacity was improved from low to medium or high. The synbiotic intervention in the study included multi-species probiotics with *L*. *acidophilus*, *L*. *plantarum*, *L*. *rhamnosus*, *Bifidobacterium infantis*, and *S*. *thermophilus*. No explicit mention of the prebiotic component could be discerned. However, in the discussion section of the article, the study authors discussed saccharides–inulin and fructans which could possibly be the prebiotic component of the synbiotic intervention while the effect of prebiotic on probiotic was unexplained. The contributors of the study concluded that daily administration of synbiotic decreases salivary viscosity and increases buffering capacity of the saliva.

### Arginine and probiotics as synbiotics

A very recently published *in vitro* study [39] highlights the possibility of L-arginine and *L*. *rhamnosus* GG synbiotic for caries prevention. The authors of the study examined the effect of L-arginine on the growth of *L*. *rhamnosus* GG and the combined L-arginine and *L*. *rhamnosus* GG on the growth of *S*. *mutans* ATCC 700610 (UA159). The probiotic *L*. *rhamnosus* GG was derived from a commercial probiotic formulation Culturelle (i-Health, Inc., Cromwell, USA) and was found to match the *L*. *rhamnosus* ATCC 53103 strain. The effect of prebiotic arginine on the growth of probiotic *L*. *rhamnosus* GG was examined using metabolic activity assessment indicative of bacterial viability with qualitative analysis by confocal laser scanning microscopy. Further, the effect of synbiotic on the growth of pathogenic *S*. *mutans* was estimated using conventional CFU counting, determination of biofilm biomass, qualitative evaluation of biofilm thickness using confocal imaging, and a molecular method using real-time qPCR. Biochemical assessments were performed to determine spent media pH and lactic acid production immediately after treatment and 24-h post-treatment when incubated in an anaerobic chamber. The study results showed that the increasing concentrations of L-arginine enhanced the growth of the probiotic *L*. *rhamnosus* GG. The synbiotic significantly inhibited the growth of cariogenic pathogen *S*. *mutans*. The study also highlights the possible mechanistic properties by which arginine augments the growth of the probiotic *L*. *rhamnosus* GG while further ciphering a contemplated mechanism of action on a cariogenic pathogen and oral commensals. The study limitations included the use of a closed biofilm model as opposed to an open poly-microbial model which can be explored through further studies.

A narrative review [36] summarized the current evidence on the role of oral prebiotics and probiotics in caries prevention and caries management. The review comprised two main sections–one on the role of oral prebiotics and the other on oral probiotics. In the first section, the role of urea (carbamide) and arginine as prebiotic supplements in caries prevention were discussed. Based on the included primary studies, the review found that urea supplementation might lead to calculus formation with limited evidence on caries inhibition. Conversely, arginine was found to be as a promising caries-inhibiting prebiotic, but with concerns on bias of the clinical trials due to involvement of the industry.

The second section reported a favourable role of probiotics for caries prevention. However, the clinical trials reviewed highlighted problems with blinding, attrition, selection bias and short study duration. The clinical studies also differed in use of intervention species and strains as probiotics, leading to additional limitations for data synthesis. The review also emphasized the need to assess the cost-effectiveness of probiotic therapy, which had not yet been reported in the literature. Given the weaknesses of the reported evidence, the study concluded that probiotics might serve as an adjunct to an individual’s established preventive practice.

In summary, the review authors suggested that the combination of oral prebiotics (arginine) and recently isolated probiotic arginolytic strains (*Streptococcus dentisani* and *Streptococcus A12*) show potential promise in the prevention of dental caries and present a developmental challenge for the future, this reaffirms the recommendations from another review [44], which stated that the combination of arginine and ADS (Arginine Deiminase System)-positive probiotics might constitute a non-pharmacological advancement for caries prevention. Further development and evaluation of such a combination needs to be explored.

Currently, the evidence on the role of synbiotics in caries prevention is relatively weak as only *in vitro* studies, a quasi-experimental clinical study, and a narrative review were found to address this specific topic. Data synthesis could not be performed as no controlled clinical studies were identified.

## Discussion

According to the search results, this is the first review on the effect of synbiotics in caries prevention. Results of the present review show that most of the identified potential synbiotics for caries prevention are still in their laboratory or pre-clinical stages of investigations with no controlled clinical studies or animal studies reported. The potential caries-preventive synbiotics that have been tested thus far include saccharides (prebiotic) and lactobacillus spp. or lactobacillus spp. including multi-species probiotics; whereas only 1 *in vitro* study explored the possibility of an arginine-based synbiotics. Moreover, no synbiotic consumer product for caries prevention could be identified in the literature, highlighting the need for commercial development, but with care to determine its cost-effectiveness *versus* beneficial effects. Therefore, it can be summarized that the use of synbiotics for caries prevention is still in its inceptive stages of investigation, which is evident from the results of the present review with all the included articles being published in the recent decade (2010 onwards).

It is noteworthy that 2 [35,37] out of 5 [34,35,37,38] *in vitro* studies were preliminary reports with one concluding that GOS did not enhance the function of *L*. *acidophilus* to inhibit the growth of *S*. *mutans* [35]. The result of the preliminary report on GOS was in agreement with another study [34] that also showed no effect on the growth rate of *L*. *acidophilus*, in both individually cultured or co-cultured media, with all concentrations of GOS and FOS as compared to the control. However, the growth rate of *S*. *mutans* was significantly reduced when co-cultured with *L*. *acidophilus* and the proper concentrations of prebiotics (GOS and FOS) [34]. In the study of GOS or FOS prebiotics, the medium used for bacterial growth was MRS broth, a suitable growth medium for both *S*. *mutans* and *L*. *acidophilus*. However, despite the use of suitable growth medium, the growth of *L*. *acidophilus* ATCC 4356 was not substantiated in the presence of the saccharide prebiotics. According to the definition of synbiotics, it can be inferred that, although the combination has a synergistically inhibitory effect on the growth of *S*. *mutans*, which could be due to the inhibitory potential of the individual components; the proposed GOS or FOS prebiotic-based synbiotics do not fulfil the definition of synbiotics as they did not enhance the growth of *L*. *acidophilus* (probiotic).

Another preliminary report [37] on synbiotics that utilized GMH-based prebiotics showed that *L*. *acidophilus* probiotics outgrew pathogenic *S*. *mutans* in the mixed cultures when supplemented with 2% GMH. However, the study did not explicitly explain the effect of GMH supplemented to *L*. *acidophilus* compared to the control. Hence, further studies are needed to resolve the effect of GMH on *L*. *acidophilus* probiotic using advanced molecular methods, in addition to the conventional techniques used in the preliminary study, before its eligibility as a potential prebiotic can be determined.

Similar to the above discussed preliminary report, the study [38] exploring the prebiotic and probiotic effects of 12 saccharides and 40 lactobacilli spp. showed that all lactobacilli spp. exhibited growth with all 12 saccharides. However, the comparative growth of the investigated lactobacilli spp. with/without saccharide prebiotics was not explicitly shown. Thus, similar to GMH, any of the 12 saccharides with 40 lactobacilli spp. as a combined synbiotic remains unconfirmed. Nonetheless, the study highlighted the potential use of probiotic culture supernatant, in addition to the live probiotic, for inhibition of insoluble glucan production activity of *S*. *mutans*. This suggests that metabolites or by-products of the bacteria, rather than the presence of the bacteria themselves, are responsible for the inhibitory effect. Ultimately, the study identified 5 potential probiotics and 3 prebiotics that inhibited the activities and growth of *S*. *mutans*. Further studies are needed to examine the combined effect of prebiotics—xylose, xylitol, and arabinose with *L*. *gasseri*, *L*. *fermentum*, *L*. *paracasei*, *L*. *plantarum*, and 120. *Lactobacillus* spp. as synbiotics and the actions of individual synbiotic against the cariogenic pathogen *S*. *mutans*.

Only 1 clinical trial [40] explored the effect of multi-species probiotics likely combined with saccharides prebiotics as a synbiotic (commercial product for gut microflora) for caries prevention using salivary viscosity and buffer capacity as biomarkers. The study duration was limited to 6-day; however, still significant changes were determined between pre- and post-treatment outcome measures. Being a quasi-experimental clinical trial, no control arm was included and thus, limited controlled observations to derive adequate evidence. Another limitation of the study was that the subject recruitment was based on consecutive-convenience sampling which excludes a randomization component. Also, the mechanistic properties of the synbiotics were not clearly identified while additional constraints of mechanistic understandings were implied since the prebiotic component in the synbiotic intervention was not explicitly mentioned. Therefore, referring to the intervention as a synbiotic remains unclear.

The included narrative review suggests the development of an arginine-based synbiotic with arginolytic bacteria, *Streptococcus dentisani and Streptococcus A12*, for the prevention and management of dental caries, though no studies on arginine-based synbiotic formulations could be identified for inclusion in the current scoping review. Based on several *in vitro* studies [45–48] and clinical trials [49–51], arginine has been shown to have potential prebiotic effect against cariogenic microcosms; as it is known to improve pH and enhance ecological homeostasis. Arginine increases the growth of arginolytic bacteria (*Streptococcus sanguinis*, *Streptococcus gordonii*, and *Streptococcus parasanguinis*), which can maintain ecological symbiosis and prevent microbial dysbiosis. Therefore, the inclusion of the prebiotic arginine in a synbiotic formulation represents a promising approach for the control of oral biofilm, decreasing the risk of dental caries and addressing a worldwide problem. However, this review highlights the challenges of developing a combined synbiotic formulation due to such non-existing therapy with an appropriate deliverable form. Although challenging, a recently published *in vitro* study [39] explored the possibility of arginine-based synbiotic and explained the mechanistic of the combined prebiotic and probiotic intervention to develop as a synbiotic against cariogenic pathogen. It is noteworthy, that the *in vitro* study [39] included *L*. *rhamnosus* GG probiotic for the development of an arginine-based synbiotic intervention as opposed to the suggestions in the narrative review that recommend to utilize arginolytic probiotics (*Streptococcus dentisani and Streptococcus A12*).

Taken together, the evidence for saccharides-based synbiotics for caries prevention is weak. By contrast, the potential of amino acid (esp. arginine)-based synbiotics appears promising for caries prevention. Arginine, a semi-essential amino-acid, is metabolized by arginolytic bacteria to produce ornithine, citrulline, CO_2_, ATP, and ammonia; whereby ammonia production neutralizes the acids produced by the cariogenic consortia [52] with evident effects shown in clinical trials. Most probiotics supplements either contain *Lactobacillus spp*. or *Bifidobacterium spp*. or both, of which several *Lactobacillus spp*. have been shown to have a caries-preventive or inhibitory effect through clinical trials [19]. *Lactobacillus spp*. can inhibit human pathogenic oral bacteria (*S*. *mutans*, *S*. *sobrinus*, *P*. *gingivalis*), increase pathogenic bacteria colonization resistance, replenish and restore healthy microflora, and enhance immunological functions [53–57]. *Lactobacillus spp*. are also known to survive better in harsh conditions of oral cavity and gastro-intestinal tract as compared to other probiotics [58]. Further, *Lactobacillus spp*. need amino acids and peptides for their growth and cellular activities [59]. Certain lactobacilli spp. also lack the potential to synthesize amino acids for their growth and thus, require exogenous amino acid supplementation [39,60,61]. Findings of this scoping review show that all proposed synbiotics in *in vitro* studies included lactobacilli spp. with independent inhibitory effect against cariogenic *S*. *mutans*. Hence, the development of a combined amino-acid (esp. arginine) prebiotic and lactobacilli spp. probiotic as a caries-preventive synbiotic seems appropriate, due to their useful synergistic effect and low risk of complications.

The present review proposes three recommendations for future research: (i) evaluation of the combined (prebiotic and probiotic) synbiotic for its effect on cariogenic pathogens in an open/closed mono- and multi-species biofilm model, followed by high quality RCTs with adequate follow-up period, (ii) identification of the mechanisms of actions of synbiotic supplementation against cariogenic pathogens and dental caries, and (iii) determination of the efficacy of specific strains of probiotics, optimal dosage of pro- and pre-biotics, and treatment duration for future synbiotics research. Above all, the role of fluorides in caries prevention cannot be ignored as it has been the mainstay in the prevention of dental caries as evident by the decline of dental caries worldwide [62]. Hence, every attempt should be made to see that the proposed synbiotics and fluorides do not have antagonistic effects against each other, which might reduce the overall effectiveness of the individual therapy.

The main limitations of this review are: (i) the scant number of studies into the effect of synbiotics on caries prevention; (ii) the searches were limited to English language and (iii) a lack of appraisal of the quality of evidence though this is an optional element in scoping review. On the other hand, the present review addresses the major essential elements of the scoping review i.e. examining the available research, summarizing research findings, and identifying research gaps. This review does not provide any recommendations on the intervention nor establish a platform to undertake further systematic review as the concept is still in its infancy stages of development and requires high-quality controlled clinical data to provide conclusive recommendation for dissemination of the concept. The scoping review clearly identifies the need to further explore the caries-preventive effects of synbiotic to optimize their use.

## Conclusion

Based on the findings of the present review, it is concluded that: (1) Several synbiotics have been proposed for caries prevention; however, their eligibilities for classification as true synbiotics needs to be carefully addressed. (2) Due to a lack of clinical studies on synbiotics for caries prevention, evidence on their caries-preventive potential is weak. (3) Amino acid (esp. arginine)-based synbiotics appears promising for caries prevention. Future studies are needed to examine the potential synbiotic against cariogenic pathogens.

## Supporting information

S1 Table PRISMA-ScR checklist. (DOCX)

S2 Table Database specific search strategy. (DOCX)
