# Supplementary material for: Synbiotics in caries prevention: A scoping review
Source: PLoS One. 2020 Aug 12;15(8):e0237547. doi: 10.1371/journal.pone.0237547 (PMC7423128; doi:10.1371/journal.pone.0237547)
Supplement: S2 Table — (DOCX) [file pone.0237547.s002.docx]

**Table S2. Database specific search strategy**

| **PubMed** | (synbiotic[All Fields] OR synbiotic'[All Fields] OR synbiotic's[All Fields] OR synbiotic2000[All Fields] OR synbiotic2000trade[All Fields] OR synbiotically[All Fields] OR synbiotics[All Fields] OR synbiotics'[All Fields] OR synbioticum[All Fields]) AND ("dental caries"[MeSH Terms] OR ("dental"[All Fields] AND "caries"[All Fields]) OR "dental caries"[All Fields] OR "caries"[All Fields]) |
| --- | --- |
| **Scopus** | TITLE-ABS-KEY ( ( synbiotic* )  AND  ( caries ) ) |
| **WoS** | TOPIC: ((synbiotic*) AND (caries)) Indexes=SCI-EXPANDED, SSCI, A&HCI, CPCI-S, CPCI-SSH, ESCI Timespan=All years |
